# Assembly Line Education: A Novel Educational Technique for Today's Learners

**DOI:** 10.7759/cureus.7065

**Published:** 2020-02-21

**Authors:** Javier Rosario, David Lebowitz, Amanda L Webb, Latha Ganti, Ariel Vera, Tracy Macintosh, Ayanna Walker, Jose Rubero

**Affiliations:** 1 Emergency Medicine, University of Central Florida College of Medicine, Orlando, USA; 2 Emergency Medicine, University of Central Florida College of Medicine/Hospital Corporation of America Graduate Medical Education Consortium of Greater Orlando, Orlando, USA; 3 Emergency Medicine, Envision Physician Services, Orlando, USA; 4 Emergency Medicine, Polk County Fire Rescue, Bartow, USA; 5 Emergency Medicine, University of Central Florida College of Medicine/Hospital Corporation of America Graduate Medical Education Consortium of Greater Orlando, Kissimmee, USA; 6 Emergency Medicine, Osceola Regional Medical Center, Kissimmee, USA; 7 Emergency Medicine, Osceola Regional Medical Center, Orlando, USA

**Keywords:** medical education, emergency medicine, simulation

## Abstract

Background

Education is undergoing a transformation. The traditional passive lectures are failing to capture and inspire the new generation of learners who value more active and collaborative learning techniques.

Objective

We sought to create a novel educational technique to integrate into our curriculum that would be more personalized, employ more active learning and collaboration, and allow for an effective assessment of resident strengths and weaknesses.

Discussion

We created a monthly assembly line education academic half-day that evolved to replace one of the typical in-classroom didactics each month. Faculty run small-group simulation rooms, procedure workshops, competitive ultrasound, and wellness stations through which residents and medical students rotate.

Conclusion

This novel education technique resulted in a more personalized approach that increased resident interest, sparked the creation of a very popular MedEd-Simulation elective, and allowed the faculty to gain a better sense of resident strengths and deficiencies.

## Introduction

Education is going through a transformation, where we are finding that passive lectures and traditional methods of learning and teaching are leading to boredom, sleeping, distraction, or just inattention. It is clear that the millennial residents and learners of today have different learning styles compared with other learners. The millennial generation, born between 1980 and 1990, is frequently described as impatient as they expect instant gratification and access to information, use of technology, and the need for frequent praise and feedback. They tend to be team-oriented and collaborative, but they want information tailored specifically to them, and traditional classroom lecturing does not meet the need for this level of engagement [1-2].

According to a study by Knight and Wood, students’ understanding of concepts was more effective when they were actively engaged in learning rather than in passive lecture environments. This same study also found that problem-solving skills of those in a more interactive classroom setting improved when compared with those in a lecture-based class [3]. Additional benefits of this type of environment include student accountability, content mastery, development of critical thinking skills, improvement of interpersonal skills, and improved professionalism [4].

Throughout graduate and undergraduate medical education, we see a shift away from formal podium-based lectures and toward more interactive and engaging modes of teaching. The flipped classroom model has gained significant popularity over the past decade as it allows technophiles to access and explore materials beforehand, allowing time spent as a group facilitated by expert faculty to be less focused on basic concepts, but instead about solving problems, exploring controversies, and mastering new knowledge [5]. Simulation in medical education has been cited as an effective and often-used tool in medical education [6].

Gamification refers to using game design elements in an effective way to augment the traditional medical education approach to patient safety and competency-based education in a way that limits learner burnout while creating a sense of fun that promotes ongoing participation and engagement [7-8]. Several forms of gamification reported in the literature include using social media platforms such as Twitter, Kahoot!, ‘escape rooms’, team-based skills, ultrasound, and trivia competitions [9-14]. Another published method of increasing learner engagement and retention is for senior residents to collaborate with faculty to create interactive workshops and shape curriculum development [15]. Although, to our knowledge, the concept of an assembly line education (ALE), where all participants in small groups rotate through simulation, procedure, workshop, competitive ultrasound, and small group discussion stations as a regular part of didactics has not been published.

## Materials and methods

This study took place within the University of Central Florida's College of Medicine's Clinical Skills and Simulation Center. This 7,500 square foot state-of-the-art facility provides a variety of clinical settings for students to gain hands-on experience in order to learn and practice essential skills. There are 16 exam rooms that are set up with the medical equipment found in a standard clinic room or emergency room bay. All rooms are video-compatible and have an instructor’s one-way glass-viewing station.

We sought to create and integrate a novel education technique tailored to a new generation and learning style of residents and medical students. This technique employs more active learning, small group collaboration and (later) competition, and immediate feedback. Our goals were to increase resident engagement while allowing faculty to more effectively assess and address resident strengths and deficiencies.

## Results

**Figure 1:**
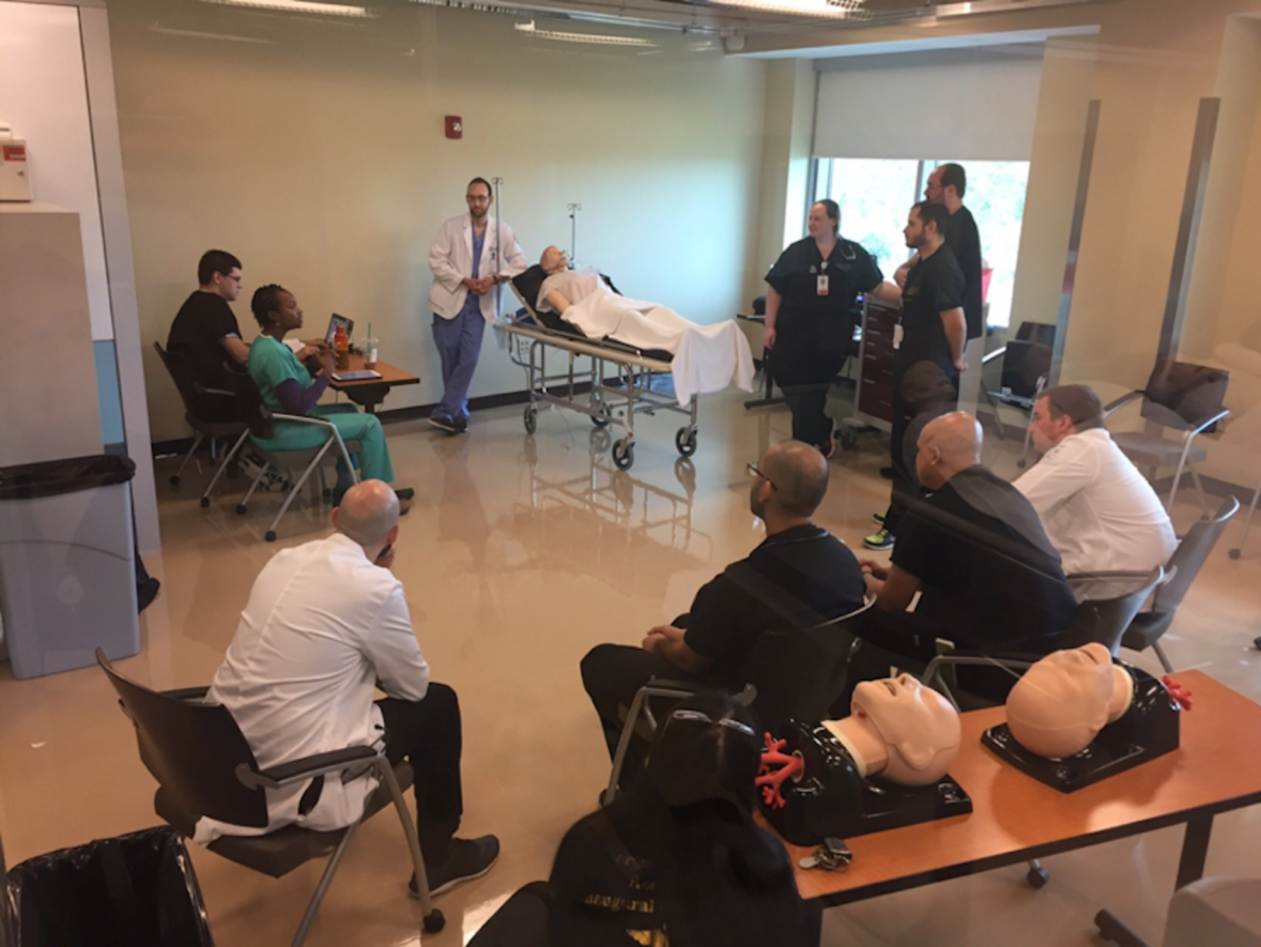
Simulation classroom

We started by adding simulation to our curriculum, with a monthly group simulation event that would replace a typical in-classroom didactic (Figure 1).

**Figure 2:**
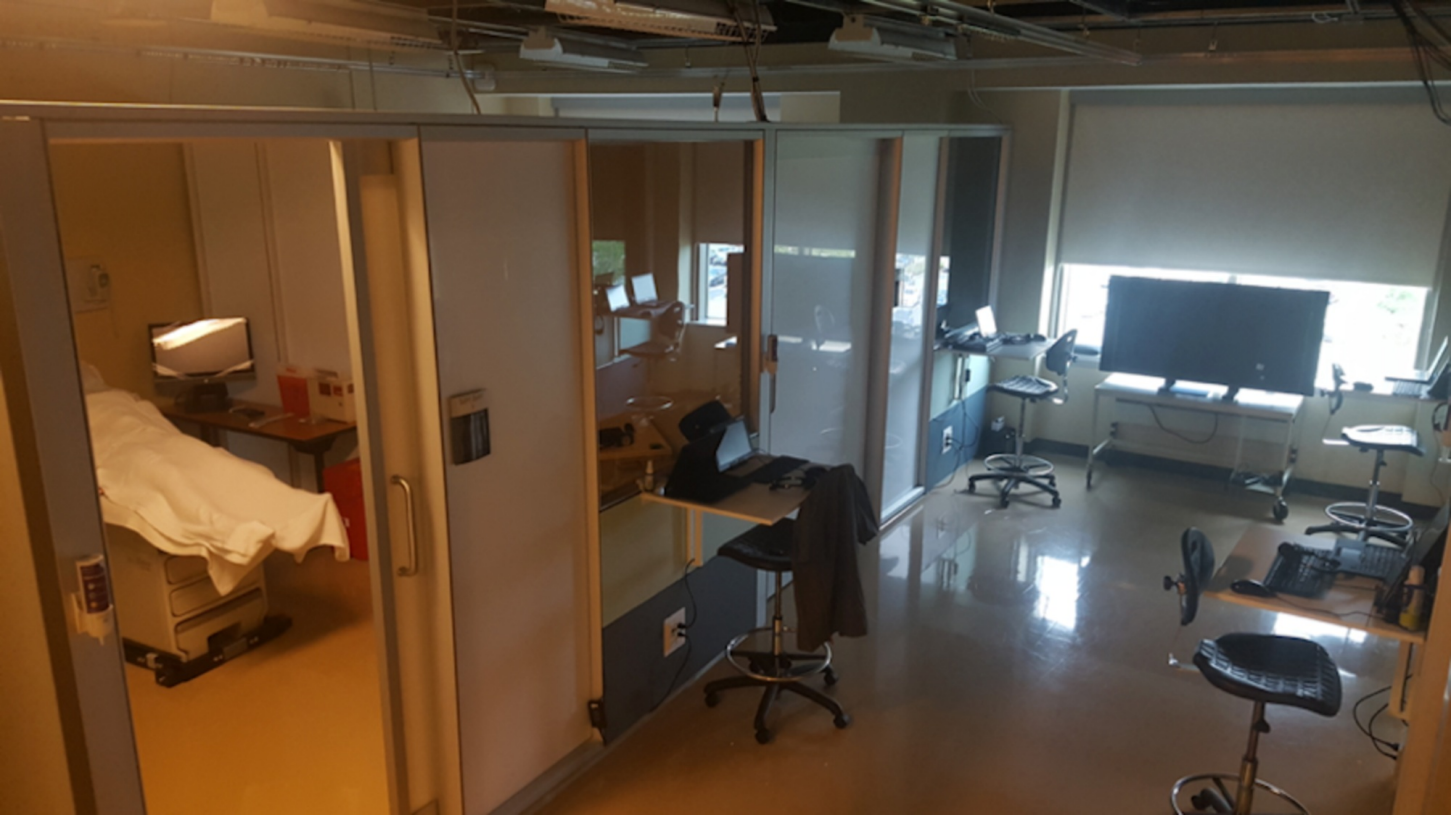
Remodeled simulation setup

Soon after implementing, we realized that this approach was too impersonal and led to a poor assessment of resident strengths and weaknesses. We also feared that with this approach, residents would feel too much pressure from their peers. After several months of discussion with our sponsoring college of medicine, we submitted feedback and recommendations, which included a complete remodel of the simulation center (Figure 2).

**Figure 3:**
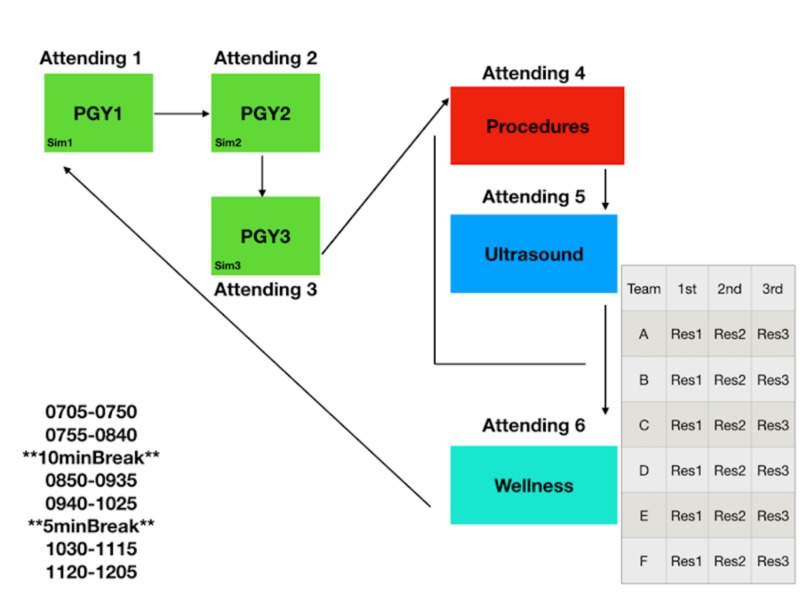
Assembly line education algorithm PGY, post-graduate year; Res, resident

With this new setup, we gained four simulation bays, one large debriefing or small group room, and an additional flex/dark room. These changes led to the creation of ALE (Figure 3).

**Figure 4:**
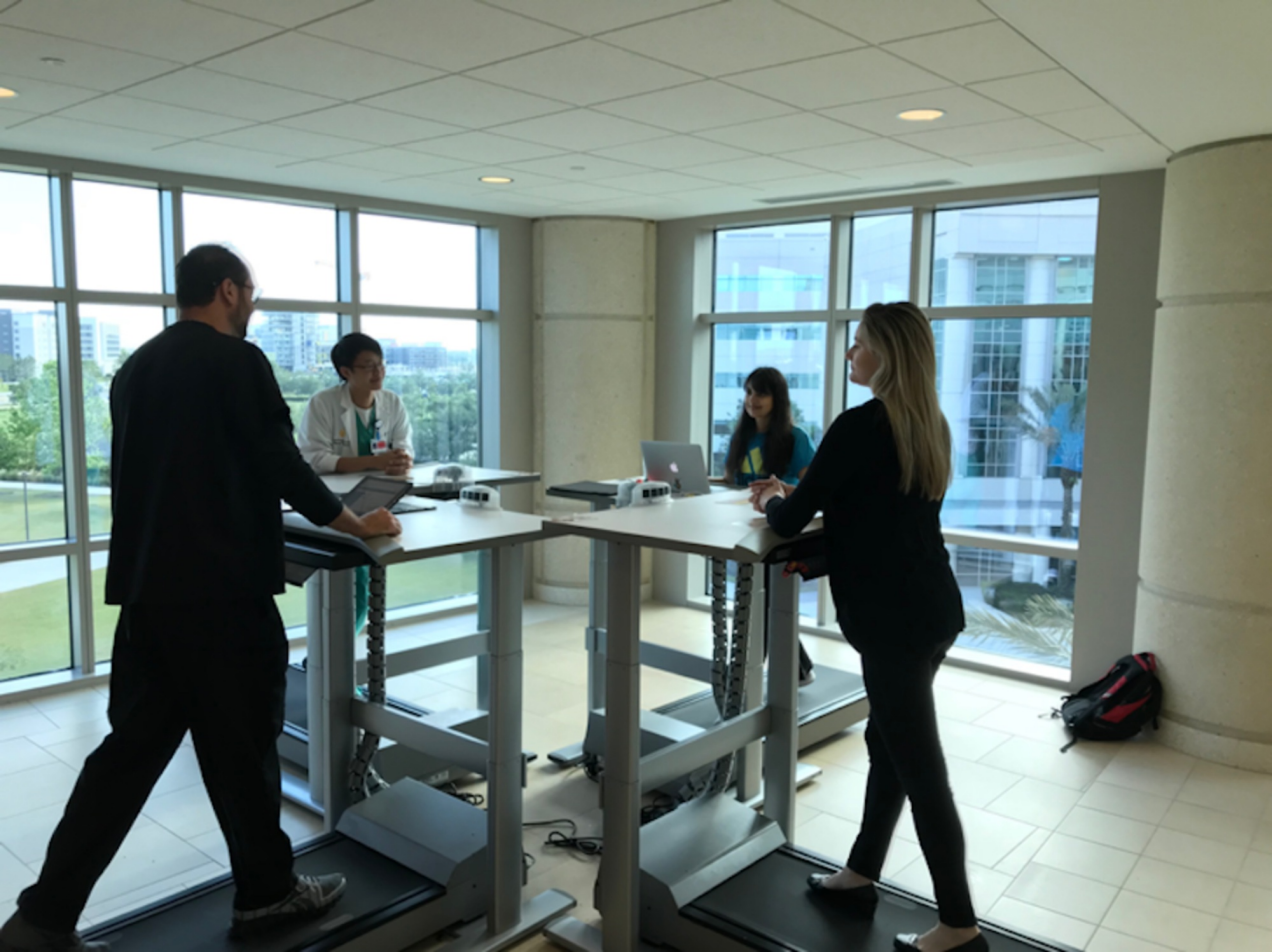
Wellness session on treadmills

**Figure 5:**
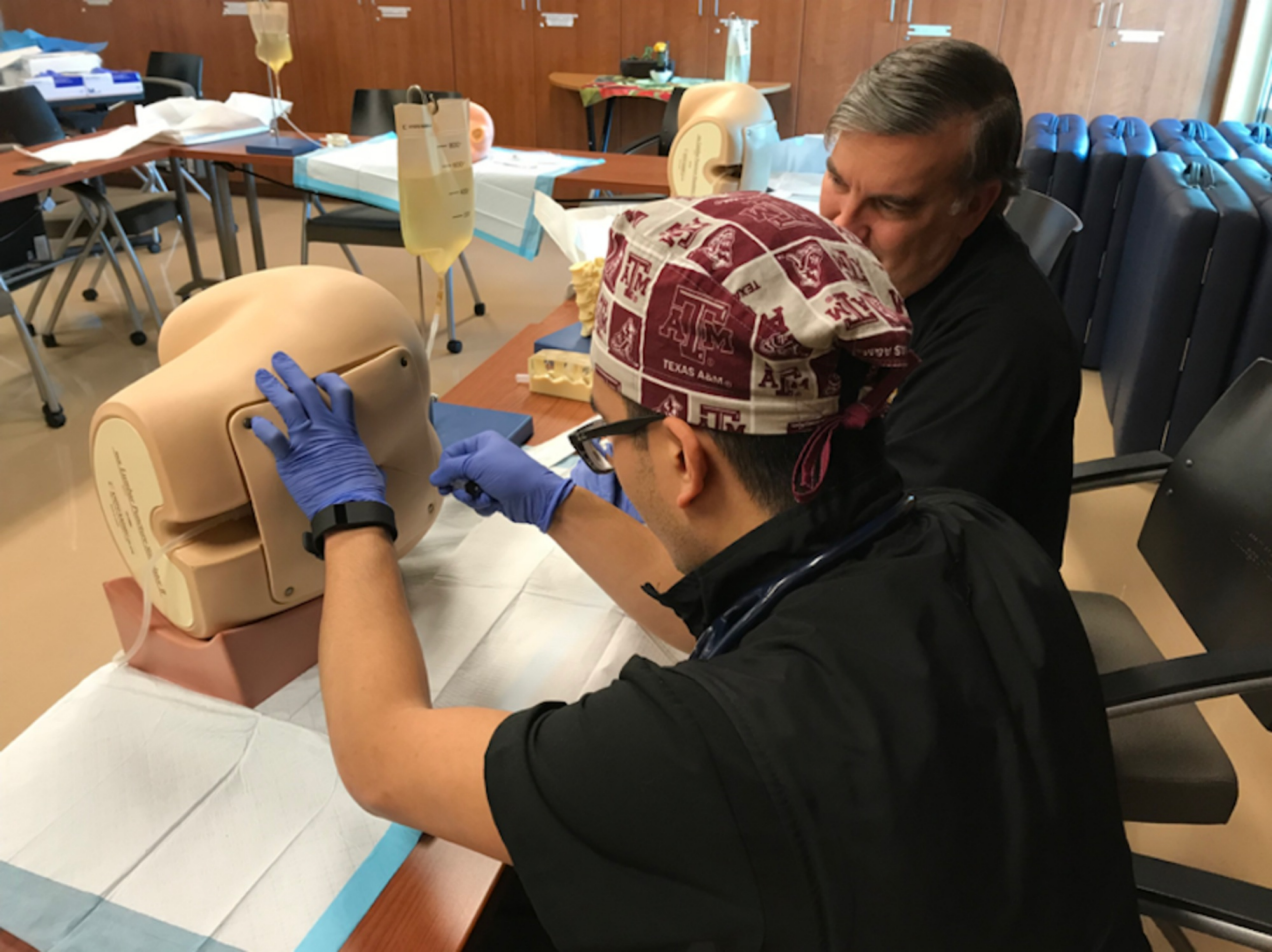
Procedure simulation station

**Figure 6:**
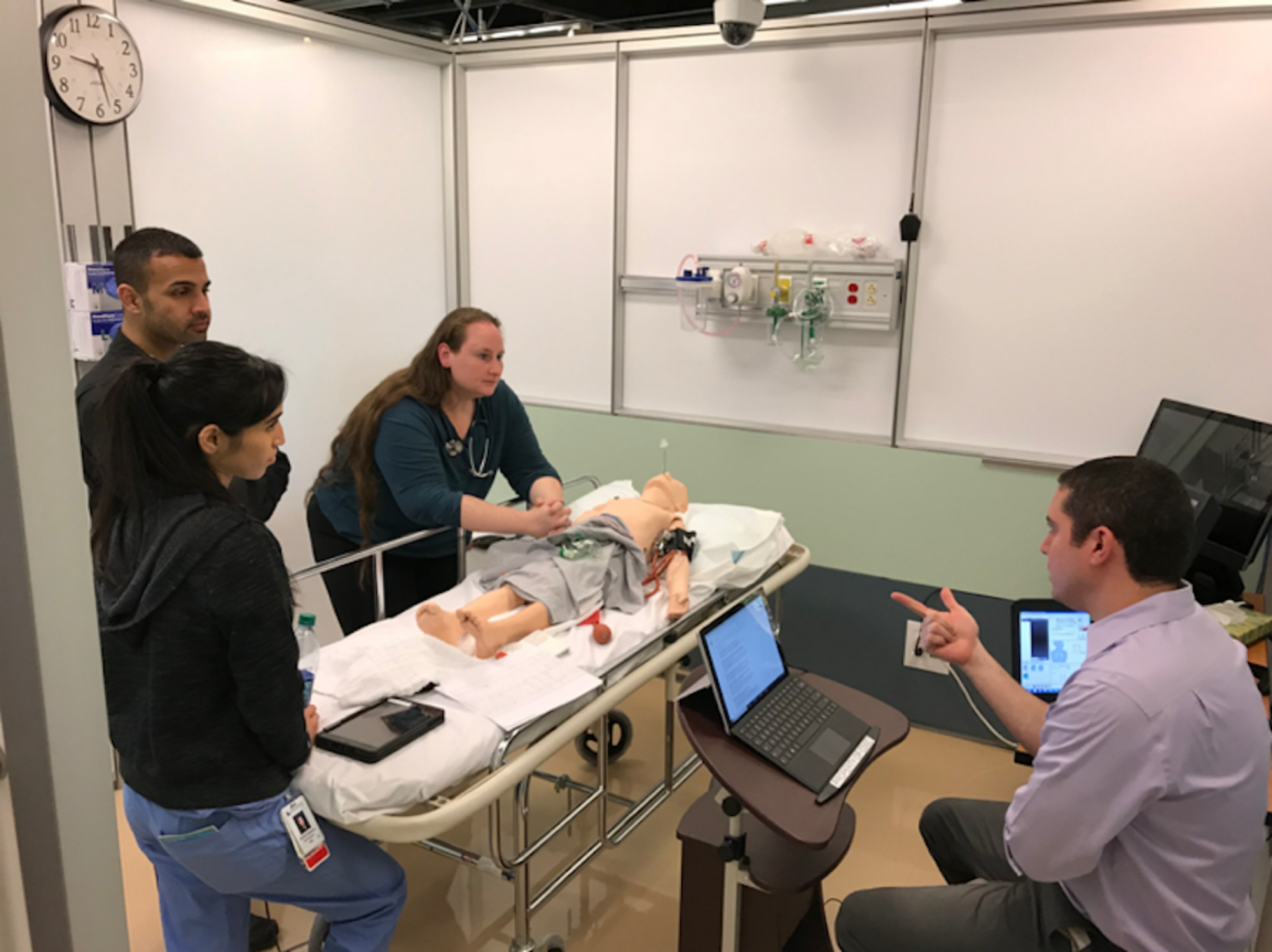
Pediatric simulation scenario

The ALE model has led to a more personalized approach for each resident. They have shown improved willingness to participate, ask questions, and discuss how they feel. With this model, faculty are able to better assess and address resident strengths and deficiencies in real time. For the model to work, the faculty remain stationary, and the residents move around to the different stations (as in an assembly/production line). Each team consists of one third-year resident, one second-year resident, and one first-year resident, which may also substitute or include an off-service resident or medical student. Each small group session (on the right side of Figure 3) can be interchanged into whatever needs to be evaluated or into any small group discussion that is of interest (Figures 4-6).

For communication during the event, each station uses a group messaging platform (Slack®, Slack Technologies, San Francisco, CA) to send residents information and visual stimuli (electrocardiograms, labs, imaging) pertaining to their ongoing case or scenario. Messages and stimuli are controlled by faculty or persons running and leading the case. Creating cases can be a challenge. When necessary, we will use open-access internet platforms, such as emsimcases.com and thesimtech.com, for case selection. Tapping into the competitive nature of physicians, the ultrasound module occasionally creates friendly competition between small groups that has positively affected resident engagement.

Additionally, the ALE helped stimulate resident interest as well as the creation of a very popular MedEd-Simulation elective. As part of the elective, the senior resident collaborates with faculty to create a simulation scenario that the resident runs during the ALE that coincides with their elective.

## Discussion

In the near future, we hope to keep improving the ALE and to add other aspects of emergency medicine into our model. We plan on implementing standardized patient, multi-specialty, and multi-disciplinary simulations. We also plan to craft social situation simulations such as against medical advice controversies, physical or mental abuse, and implicit bias. There are several challenges we have encountered during the development and implementation of ALE (Table 1).

**Table 1:** Challenges encountered during the implementation of assembly line education

Implementation challenges
Adjusting to the simulation center calendar availability
Difficulties in creating different and challenging cases on a monthly basis
Keeping track of, and logging, cases created and used previously
Limitations on the fidelity of the equipment
Faculty contribution/buy-in
Non-faculty health providers buy-in (nurses, paramedics, techs, etc.)
Faculty adherence to time limits
Inclusion of medical students and clinical rotators without being intrusive

## Conclusions

In the authors' experience, an ALE model, where participants rotate through simulation, procedure, workshop, competitive ultrasound, and small group discussion stations, has been a well-received educational innovation that could pave the way for further innovations.
